# Associations between sedentary time, physical activity and bone health among older people using compositional data analysis

**DOI:** 10.1371/journal.pone.0206013

**Published:** 2018-10-22

**Authors:** Irene Rodríguez-Gómez, Asier Mañas, José Losa-Reyna, Leocadio Rodríguez-Mañas, Sebastien F. M. Chastin, Luis M. Alegre, Francisco J. García-García, Ignacio Ara

**Affiliations:** 1 CIBER of Frailty and Healthy Aging (CIBERFES), Madrid, Spain; 2 GENUD Toledo Research Group, University of Castilla-La Mancha, Toledo, Spain; 3 Geriatric Department, Virgen del Valle Hospital, Toledo, Spain; 4 Geriatric Department, University Hospital of Getafe, Getafe, Spain; 5 Glasgow Caledonian University, School of Health and Life Sciences, Glasgow, United Kingdom; 6 Ghent University, Department Movement and Sport Sciences, Ghent, Belgium; Charles P. Darby Children’s Research Institute, 173 Ashley Avenue, Charleston, SC 29425, UNITED STATES

## Abstract

**Introduction:**

Aging is associated with a progressive decrease in bone mass (BM), and being physical active is one of the main strategies to combat this continuous loss. Nonetheless, because daily time is limited, time spent on each movement behavior is co-dependent. The aim of this study was to determine the relationship between BM and movement behaviors in elderly people using compositional data analysis.

**Methods:**

We analyzed 871 older people [395 men (76.9±5.3y) and 476 women (76.7±4.7y)]. Time spent in sedentary behavior (SB), light physical activity (LPA) and moderate-to-vigorous physical activity (MVPA), was assessed using accelerometry. BM was determined by bone densitometry (DXA). The sample was divided according to sex and bone health indicators.

**Results:**

The combined effect of all movement behaviors (PA and SB) was significantly associated with whole body, leg and femoral region BM in the whole sample (*p*≤0.05), with leg and pelvic BM (*p*<0.05) in men and, with whole body, arm and leg BM (*p*<0.05) in women. In men, arm and pelvic BM were negatively associated with SB and whole body, pelvic and leg BM were positively associated with MVPA (*p*≤0.05). In women, whole body and leg BM were positively associated with SB. Arm and whole body BM were positively associated and leg BM was negatively associated with LPA and arm BM was negatively associated with MVPA (*p*≤0.05). Women without bone fractures spent less time in SB and more in LPA and MVPA than the subgroup with bone fractures.

**Conclusion:**

We identified that the positive effect of MVPA relative to the other behaviors on bone mass is the strongest overall effect in men. Furthermore, women might decrease bone fracture risk through PA increase and SB reduction, despite the fact that no clear benefits of PA for bone mass were found.

## Introduction

Reduced bone mass is a well-known consequence of the aging process, which is largely determined by heredity and a number of factors including lifestyle, nutrition, physical activity (PA), smoking, alcohol consumption and chronic disease conditions and medication [1, 2]. Aging brings several physiological changes that lead to diseases such as osteoporosis. This pathology increases the risk of bone fracture, which can lead to decreased quality of life, disability, institutionalization, and excess mortality, making this disease an important contributor to the public health burden [3]. This condition is more common in women than in men, by a ratio of about 6 to 1 [4]. In fact, it is estimated that 6% of women aged 50–54 years have osteoporosis, and almost 50% of women over 80 years have bone mineral density (BMD) values classified as osteoporosis [5]. Therefore, over 200 million people worldwide suffer from this pathology [6]. At present, this is especially important given that the annual age-adjusted incidence of hip fracture has recently been reported to range from 58 to 574 fractures per 100,000 among women and from 35 to 290 fractures per 100,000 among men [7]; given that, poor bone health is a risk factor for bone fracture. Nevertheless, even if the incidence declines, the number of hip fractures is likely to grow as a result of increasing life expectancy and a larger number of elderly people in society [8, 9]. Despite these projections, bone health remains relatively understudied in this specific population [10].

Promotion of PA is highly recommended in the treatment and prevention of osteoporosis, together with fall prevention, nutritional supplements and pharmacological therapy [11, 12]; furthermore, recent research has suggested the efficacy of exercise in older adults as a way to reduce the risk of fractures [13, 14]. However, for a real understanding of the relation between PA and bone mass, it is necessary to know how the time spent in PA is distributed. As time within a 24-h period is finite, time spent in different movement behaviors are intrinsically collinear and co-dependent [15, 16]; as more time spent in one behavior necessarily decreases the time spent in another behavior. The waking hours are made up of a sequence of periods of sedentary behaviors (SB), light physical activity (LPA) and moderate-to-vigorous physical activity (MVPA). For this reason, we need to use a different model such as compositional analysis, a method that allows us to deal directly with the fundamental nature of movement behavior data, which are intrinsically compositional [15, 16]. Moreover, compositional analysis eliminates collinearity problems and deals with the co-dependence between time spent in different movement behaviors [15, 17].

To our knowledge no study has used compositional analysis to examine the associations between the different behaviors and bone health in older people (>65 years). Therefore, the aims of this study were: 1) to examine the relationship between movement behaviors and bone health in older people; and 2) to identify the movement behavior profile associated with bone health status and bone fracture independently.

## Subjects and methods

### Study sample and design

We selected all subjects who had been assessed with dual energy X-ray absorptiometry (DXA) and accelerometry from the Toledo Study for Healthy Aging (TSHA), a Spanish population-based prospective cohort study involving men and women over 65 years of age. The full methodology has been described previously [18, 19]. Briefly, data collection was performed in three stages. In the first one, subjects were interviewed at home by trained psychologists, the questionnaire included: socio-demographic data, activities of daily living, comorbidity, tobacco and alcohol consumption, depressive symptoms and fracture screening, in addition an extensive neuropsychological evaluation was performed for each subject and all of them completed the Mini-Nutritional Assessment questionnaire for screening of malnutrition risk [20] and a brief 14-item tool to check adherence to the Mediterranean diet [21]; finally a blood tests were performed by nurses. Then, in the second stage, a trained nurse collected information regarding anthropometrics, physical performance and clinical tests. In the final stage, bone health, body composition, PA and SB was obtained. Only the subjects that completed the three stages were included. Therefore, the sample was composed of 871 participants: 395 men (45.4%) (76.9±5.3 years) and 476 women (54.6%) (76.7±4.7 years). All data were collected from July 2012 until June 2017. The study was approved by the clinical research ethical committee of the Toledo Hospital Complex and all the subjects signed an informed consent to be included in the study.

### Anthropometrics

Anthropometric measurements were obtained on each subject immediately before DXA assessment. Both measurements were performed in the upright position, in underwear and barefoot. Height was measured in the Frankfort plane on a stadiometer with a precision of 1 mm (Seca 711, Hamburg, Germany). Body mass was determined using a balance with a 100 g precision (Seca 711, 120 Hamburg, Germany). Body mass index (BMI) was calculated as body mass divided by height squared (kg·m^−2^).

### Bone health

DXA scans were undertaken to assess BMD and bone mineral content (BMC) of whole body, lumbar spine (L1–L4), and proximal region of the femur (total hip, greater trochanter, inter trochanter, Ward´s triangle and femoral neck) using a Hologic, Discovery Series QDR densitometer (Bedford, USA). Whole body fat mass (g), lean mass (bone free) (g) and percentage body fat mass were also obtained from the total body DXA scan. All DXA scan tests were analyzed using the Physician’s Viewer, APEX System Software Version 3.1.2. (Bedford, USA). Scans were made in a supine position, wearing light clothing with no metal and no shoes or jewelry. DXA equipment was calibrated using a lumbar spine phantom and following the Hologic guidelines. The bone T-scores were calculated in each participant for the femoral neck and spine to classify by bone health status. Similarly, the number of bone fractures was also recorded from a personal interview.

### Physical activity and sedentary behaviors

PA and SB were assessed by accelerometry (ActiTrainer and ActiGraph wGT3X-BT; ActiGraph, Pensacola, FL, USA). The files were analyzed using a proprietary software program (ActiLife Pro 6). All participants were asked to wear an accelerometer on the left hip during waking hours for 7 consecutive days and remove them during any bathing or swimming activities. The delivery to and reception by the participants of the accelerometers, as well as the explanation of its use, were made personally [22]. The devices were initialized to collect data using 1-minute epochs. Non-wear time was defined as periods of at least 60 consecutive minutes of zero counts, with allowance for 2 min of counts between zero and 100 [23]. The study included only the results from participants with at least four valid days including at least 480 min (8 h/day) of wear without excessive counts (i.e., >20,000 counts) as in previous studies [24]. Each valid wearing-time minute was classified into one of the classical intensity bands using count-based threshold: SB (<1.5 METs), LPA (1.5–2.99 METs) and MVPA (≥ 3 METs). Older adult-specific cut-off points for vector magnitude (VM) counts per minute were used in this analysis [25, 26]. The total daily time spent in SB, LPA and MVPA were obtained by totaling the duration of all the bouts at each level for each day and is presented as a percentage of the waking day. The values were normalized to total wear time and averaged over the number of valid days to derive an estimate of the mean time spent in SB and each PA level per day.

### Covariates

The following information was recorded during the interview and measurement sessions: socio-demographic variables: age, gender, education (no studies, primary school completed, secondary school completed or more), marital status (single, married/living together, widowed, divorced/separated), and income (coded into 10 categories ranging from any income to >5000€/month); anthropometric and body composition variables: BMI, fat mass, lean mass; lifestyle factors considered were alcohol intake, smoking and nutritional status; health variables: frailty, thyroid disease, arthritis and calcium. These were entered in the model as covariates and retained by backward elimination if the predictor was *p*>0.2.

### Data analysis

Statistical analyses were completed using R statistical system version 3.1.1. Analyses followed the guide to compositional data analysis for PA, SB, and sleep research published by Chastin and colleagues [15]. Standard descriptive statistics (mean ± SD) were performed for sample characteristics and compositional descriptive statistics including compositional geometric means for central tendency and variation matrices for dispersion were also calculated in this study. Ternary plots with the three behaviors were generated to show the distribution of the sample compositions. The overlapped heat map allows distinguishing the areas of highest (more intense color) and lowest (less intense color) data concentration. The dispersion structure is represented by 99% and 95% normal-based probability regions around the compositional center. Likewise, compositional geometric mean bar plots of the absolute proportions of time were also generated to display the relative movement behavior profiles for bone health indicators (bone health status and bone fracture). To determine the relationship between movement behaviors and bone health, a compositional approach (CODA) based on an isometric log-ratio (ilr) data transformation was conducted to adequately adjust the models for time spent in the other behaviors. The combined effects of the relative distribution of all movement behaviors with each outcome were determined by *p*-values, with statistical significance set at *p*<0.05. The positive or negative associations between the time spent in each movement behavior and each outcome depending on the time spent in the other movement behaviors were also determined by *p*-values. Across all the CODA analyses, the models were adjusted for the previous covariates by backward elimination, with predictor retained if *p*<0.2. All statistical analyses were conducted on the whole sample and on men and women separately.

## Results

### Descriptive

**Fig 1:**
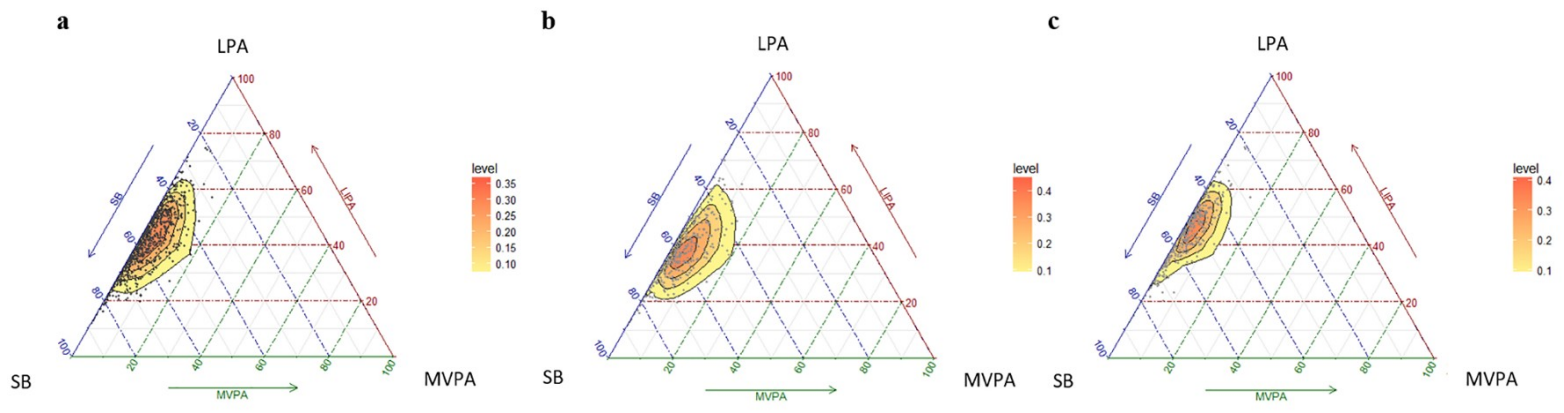
Ternary plots of the sample compositions of time spent in sedentary behavior (SB), light physical activity (LPA) and moderate-to-vigorous physical activity (MVPA) for the whole sample (a), men’s (b) and women’s (c) subgroups.

The characteristics of the participants are shown in Table 1. Of the 871 eligible subjects with the three stages completed, 776 participants were included in all analyses, 360 men (46.4%) and 416 women (53.6%). The final whole sample was decreased because of DXA scans with artefacts, e.g., metal prostheses were excluded. Similarly, subjects without enough accelerometry data (see above) were also excluded from the analysis. When comparing men and women, both showed a similar age, but men had a significantly higher body mass and height than women (*p*<0.05); nevertheless, BMI and the percentage of body fat were significantly lower in men than women (*p*<0.05). The geometric means for the minutes/day and the % of time spent in SB, LPA, MVPA for the whole sample and sex subsample are shown in Table 2. On average, older people spend 800 minutes/day in waking activities. Men spent less time in LPA and more time in SB and MVPA than women. The variability of the data is summarized in the variation matrix containing all pair-wise log-ratio variances; which is presented in Table 3. The variables with the highest co-dependence were SB and LPA in the whole sample and both subgroups. On the other hand, it can be observed that the lowest co-dependences all involved MVPA, which shows that the behaviors with the lowest co-dependence were MVPA and SB. The distribution of the sample composition is shown in Fig 1 by means of a matrix of ternary plots with three behaviors represented at the same time. Ternary plots can be understood as the scatter plots of compositions [15]. The plots reflect the fact that the highest variability is found in the direction of MVPA in the case of the whole sample and both subgroups.

**Table 1:** Anthropometric and descriptive data.

Variables	Whole Sample	Men	Women
	(n = 776)	(n = 360)	(n = 416)
Sex (%)			
Men	46.4		
Women	53.6		
Age (years)	76.8 ± 5.0	76.9 ± 5.3	76.7 ± 4.7
Body mass (kg)	73.6 ± 12.7	77.4 ± 12.0	70.2 ± 12.4*
Height (cm)	155.9 ± 9.0	162.6 ± 6.7	150.0 ± 6.2*
BMI (kg/m^2^)	30.3 ± 4.8	29.2 ± 4.0	31.2 ± 5.2*
Body fat (%)	36.6 ± 7.7	30.4 ± 5.1	42.1 ± 5.1*
Highest household educational (%)			
Less than primary school graduation	63.9	62.0	65.5
Primary school graduation	21.9	18.7	24.8
Secundary school graduation or more	14.2	19.3	9.7
Marital status (%)			
Single	5.4	7.6	3.6
Married	70.7	79.3	63.2
Widower	22.3	10.6	32.5
Separated / Divorced	1.6	2.5	0.7
Frailty (%)	2.7	2.6	2.8

Data are mean ± SD. BMI, body mass index;

* *p*<0.05, for whole sample and men vs. women.

**Table 2:** Geometric means for SB, LPA and MVPA in minutes/day and percentage of waking hours.

Sample	Minutes/day	% of waking hours
Whole Sample		
SB	450.4	56.5
LPA	323.8	40.4
MVPA	25.2	3.1
Men		
SB	470.2	59.2
LPA	295.4	36.8
MVPA	32.5	4.0
Women		
SB	433.2	54.2
LPA	348.3	43.4
MVPA	19.0	2.4

SB, sedentary behavior; LPA, light physical activity; MVPA, moderate-to-vigorous physical activity.

**Table 3:** Pair-wise log-ratio matrix for SB, LPA and MVPA.

Sample	SB	LPA	MVPA
Whole Sample			
SB	0	0.146	1.261
LPA	0.146	0	1.115
MVPA	1.261	1.115	0
Men			
SB	0	0.207	1.170
LPA	0.207	0	0.964
MVPA	1.170	0.964	0
Women			
SB	0	0.097	1.354
LIPA	0.097	0	1.257
MVPA	1.354	1.257	0

SB, sedentary behavior; LPA, light physical activity; MVPA, moderate-to-vigorous physical activity.

### Composition of the day by groups

**Fig 2:**
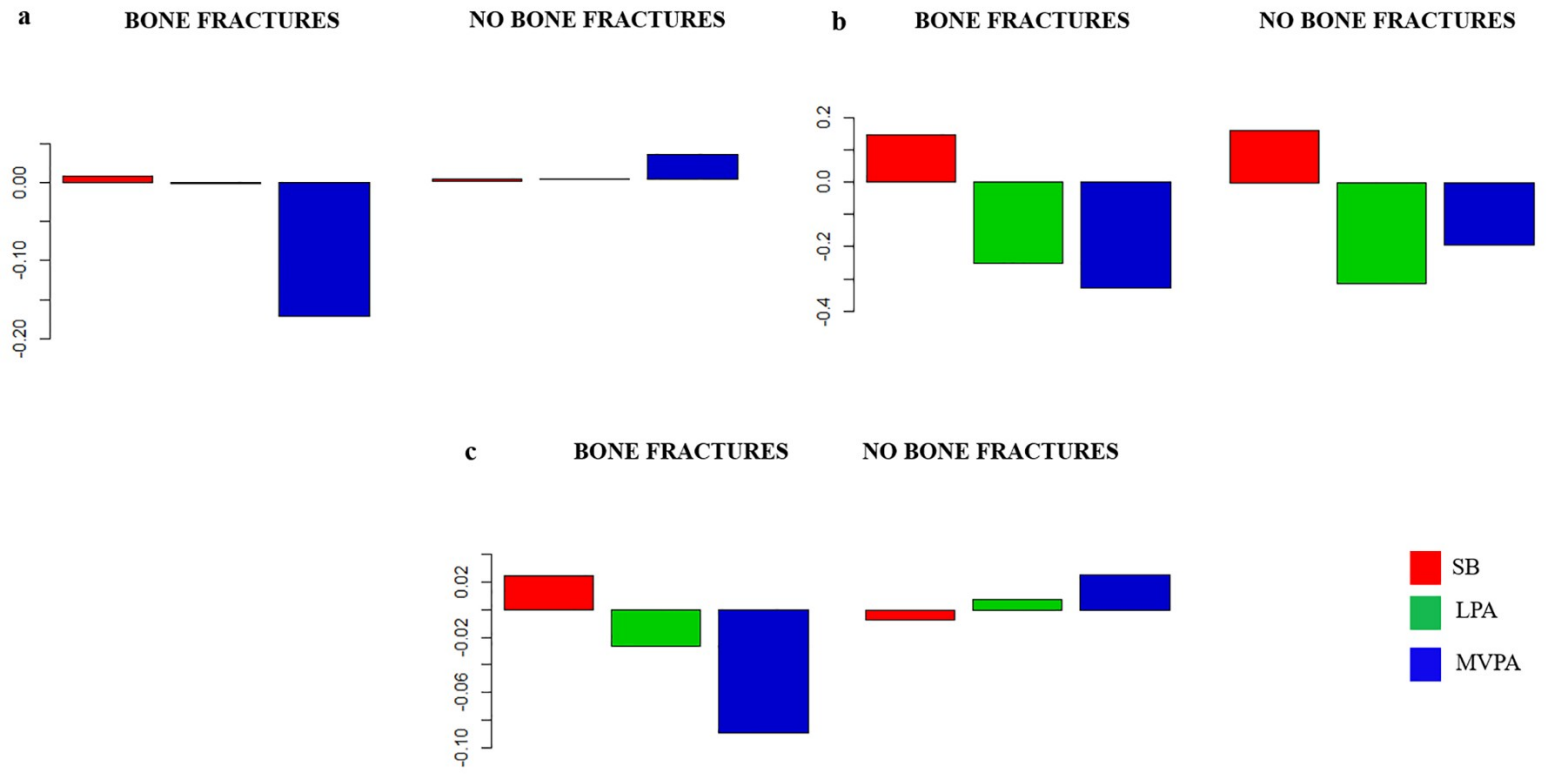
Compositional geometric mean bar plots comparing the compositional mean of the entire sample with the compositional mean of bone fracture and no bone fracture subgroups for sedentary behavior (SB), light physical activity (LPA) and moderate-to-vigorous physical activity (MVPA) for the whole sample (a), men’s (b) and women’s (c) subgroups.

**Fig 3:**
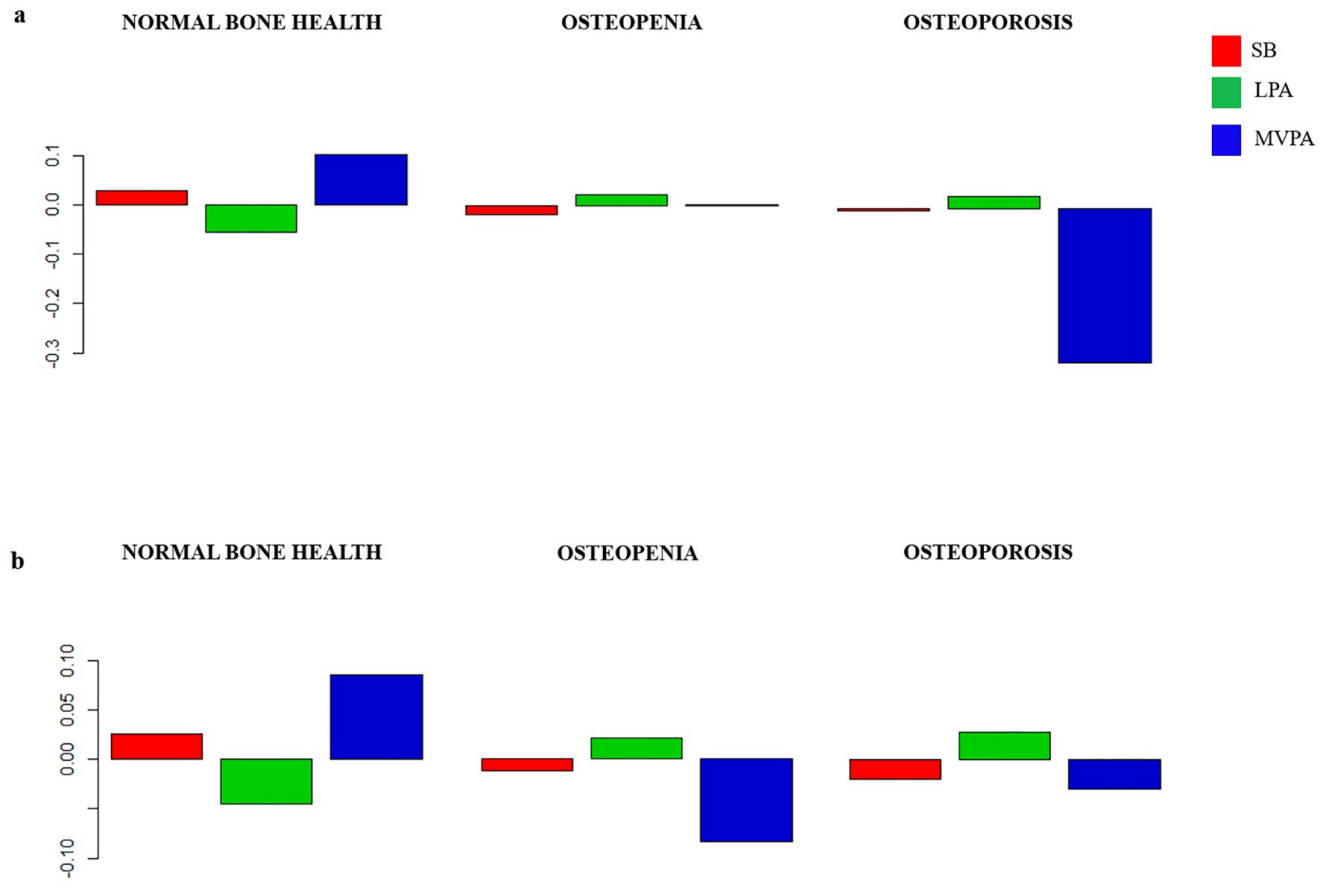
Compositional geometric mean bar plots comparing the compositional mean of the entire sample with the compositional mean of normal bone health, osteopenia and osteoporosis subgroups assessed at femoral neck (a) and at spine (b) for sedentary behavior (SB), light physical activity (LPA) and moderate-to-vigorous physical activity (MVPA) for the whole sample.

**Fig 4:**
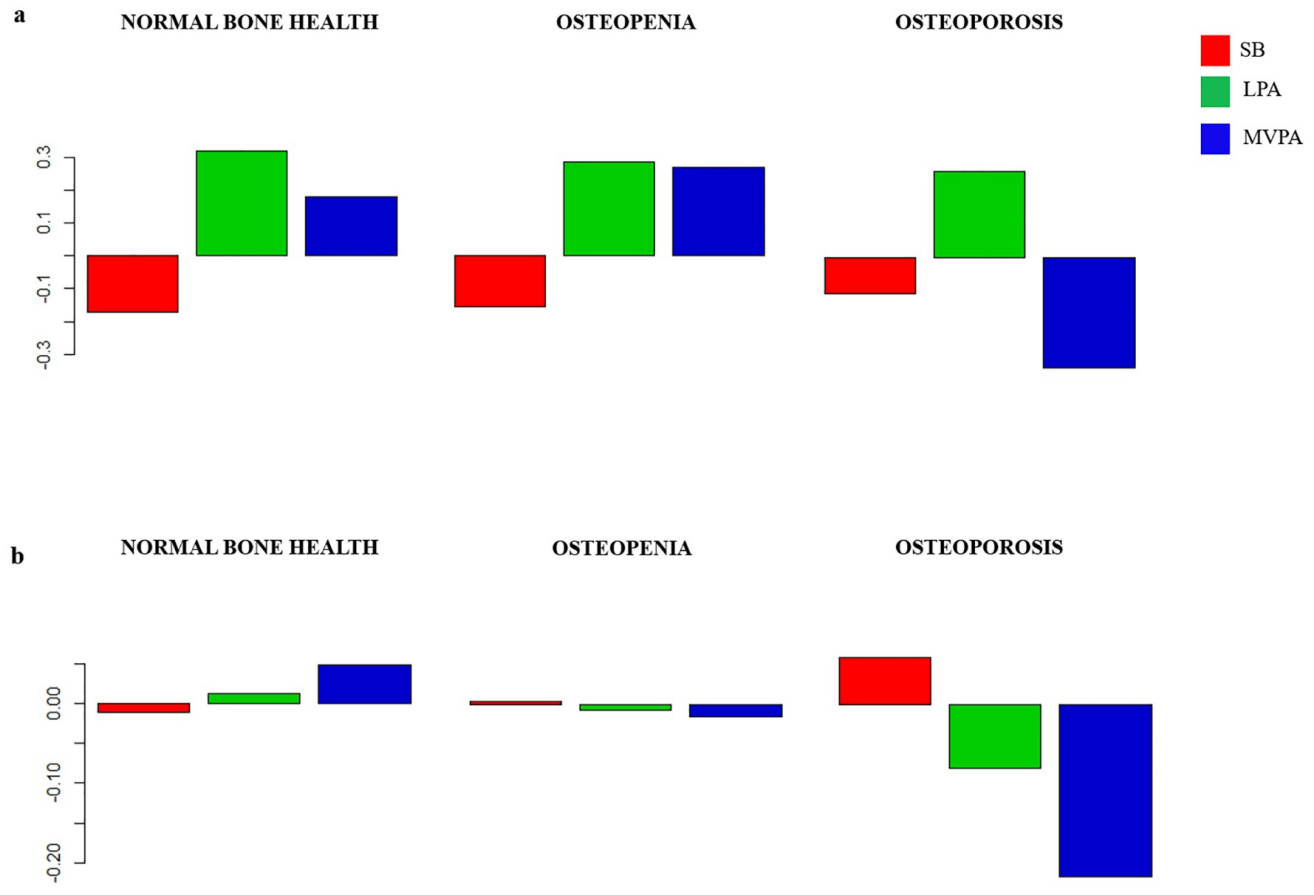
Compositional geometric mean bar plots comparing the compositional mean of the entire sample with the compositional mean of normal bone health, osteopenia and osteoporosis subgroups assessed at femoral neck (a) and at spine (b) for sedentary behavior (SB), light physical activity (LPA) and moderate-to-vigorous physical activity (MVPA) for the men’s subgroup.

**Fig 5:**
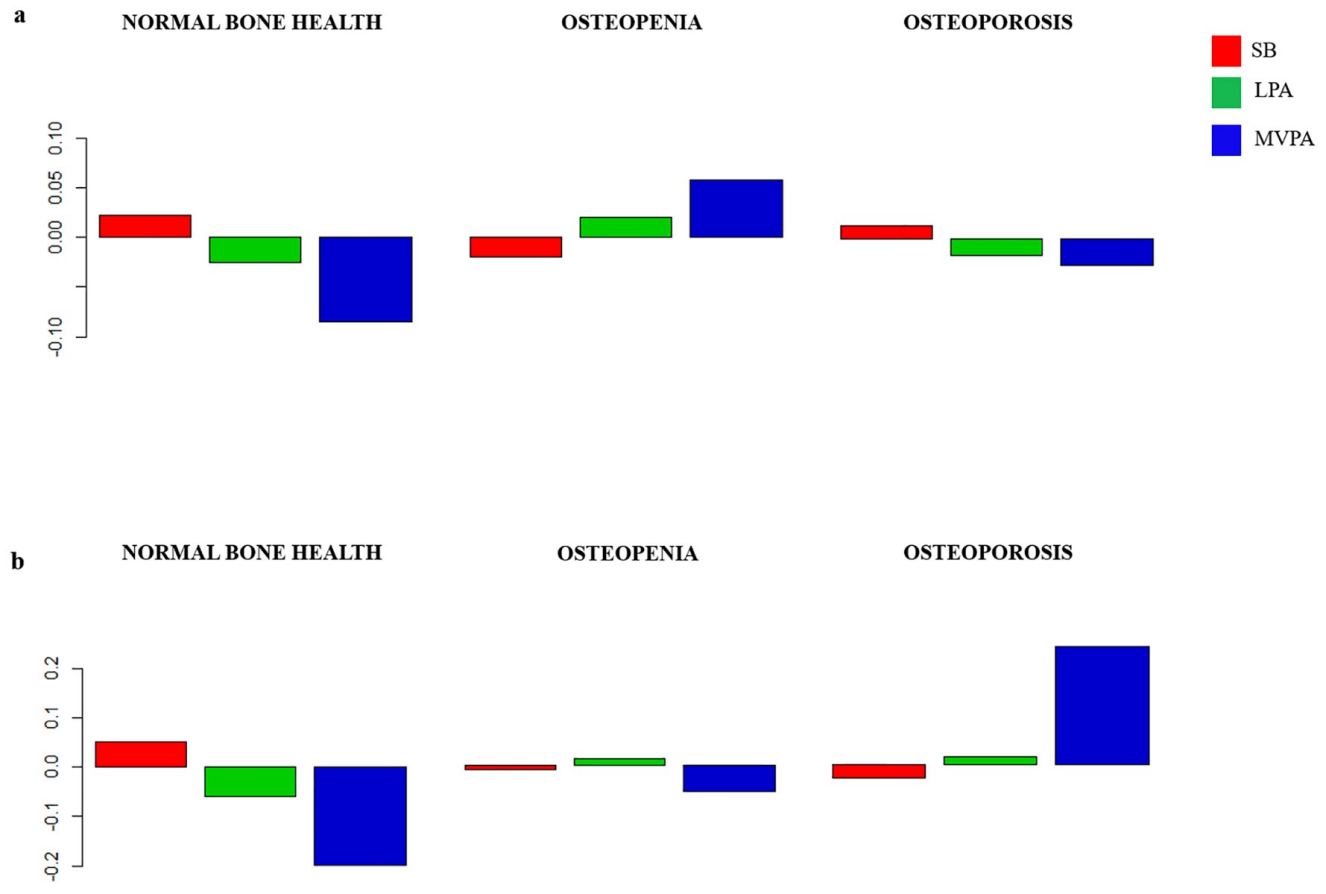
Compositional geometric mean bar plots comparing the compositional mean of the entire sample with the compositional mean of normal bone health, osteopenia and osteoporosis subgroups assessed at femoral neck (a) and at spine (b) for sedentary behavior (SB), light physical activity (LPA) and moderate-to-vigorous physical activity (MVPA) for the women’s subgroup.

The composition of the day for each group is presented as compositional mean bar plots. The whole sample, and men and women grouped by bone fractures are represented in Fig 2. Likewise, the sample grouped by bone health at femoral neck, and bone health at spine are represented in Figs 3–5, for the whole sample, and men and women, respectively. Related to bone fracture distributions, older people with bone fractures spent less time in MVPA and more time in SB compared to the entire sample. The opposite tendency was observed in the older people without bone fractures. When bone health was assessed at femoral neck, for MVPA the greatest log ratios were found in the normal bone health group and the lowest in the osteoporosis group, compared to the entire sample. A similar pattern was observed when the group was divided by bone health at spine. In men, plots are similar in the bone-fracture subgroups. In the case of the subgroups for bone health at femoral neck, older people with normal bone health presented the lowest log ratios for SB and the highest for LPA. Older men with osteoporosis showed the lowest log ratios for MVPA, while the highest log ratios in this behavior were found in the osteopenia subgroup. Older men with osteoporosis assessed at spine spent more time in SB and less time in LPA and MVPA compared to the sample average. The contrary was observed for the three behaviors in the normal bone health subgroup. In women, the proportion of time spent in SB was higher and time spent in LPA and MVPA was lower in women with bone fractures compared to the entire sample. Women without bone fractures showed the opposite pattern. When bone health was assessed both at femoral neck and spine, similar unclear patterns were found.

### Compositional data

The CODA models, which show the combined effect of the movement behaviors on each bone variable, are reported in Tables 4–6 for the whole sample, men and women, respectively. The composition of movement behaviors as a whole in the whole sample was significantly associated with leg BMC and BMD, femoral neck BMC, whole body BMD and trochanter BMD (*p*≤0.05). Time spent in SB relative to other movement behaviors was positively associated with leg BMC and BMD (*p*<0.01) and whole body BMD (*p*≤0.05), and negatively associated with arm BMC (*p*<0.05). Time spent in LPA relative to other movement behaviors was negatively associated with leg BMC and BMD and whole body BMD (*p*≤0.01), and positively associated with arm BMC (*p*<0.05). Time spent in MVPA relative to other movement behaviors was positively associated with femoral neck BMC and pelvic, leg, proximal femur and trochanter BMD (*p*≤0.05). The composition of movement behaviors as a whole in the men’s subgroup was significantly associated with leg BMC and BMD (*p*<0.05) and pelvic BMD (*p*≤0.01). Time spent in SB relative to other movement behaviors was negatively associated with arm BMC and pelvic BMD (*p*≤0.05). No significant associations were observed in the time spent in LPA relative to other movement behaviors. Time spent in MVPA relative to other movement behaviors was positively associated with leg BMC and BMD and whole body and pelvic BMD (*p*<0.05). The composition of movement behaviors as a whole in the women’s subgroup was significantly associated with whole body BMC and BMD (*p*<0.05 and *p*<0.01, respectively), leg BMC and BMD (*p*<0.01) and arm BMC (*p*<0.05). Time spent in SB relative to other movement behaviors was positively associated with whole body and leg BMC and BMD (*p*≤0.01). Time spent in LPA relative to other movement behaviors was negatively associated with whole body and leg BMC and BMD (*p*<0.05), and positively associated with arm BMC (*p*≤0.05). Time spent in MVPA relative to other movement behaviors was negatively associated with arms BMC (*p*≤0.01).

**Table 4:** Compositional behavior model for bone mass variables for the proportion of the waking hours of day spent in SB, LPA and MVPA for whole sample.

VARIABLE	MODEL *P*-VALUE	γ SB	*P*-VALUE	γ LPA	*P*-VALUE	γ MVPA	*P*-VALUE
BMC VALUES							
Whole Scan							
Whole body	0.49	8.807	0.46	-15.525	0.26	6.718	0.37
Pelvic	0.47	-2.421	0.34	1.300	0.65	1.121	0.43
Arms (mean)	0.11	**-1.889**	**0.04**	**2.158**	**0.04**	-0.268	0.62
Legs (mean)	**0.00**	**7.096**	**0.00**	**-9.650**	**0.00**	*2*.*554*	*0*.*06*
Spine							
Lumbar (mean L_1_–L_4_)	0.33	0.023	0.87	0.100	0.56	-0.119	0.14
Femoral Regions							
Proximal femur (mean)	0.60	-0.173	0.58	0.028	0.94	0.145	0.40
Femoral neck	**0.05**	-0.059	0.20	0.004	0.94	**0.055**	**0.03**
Trochanter	0.19	-0.063	0.30	0.013	0.86	0.051	0.12
Ward’s triangle	0.98	-0.001	0.91	0.000	0.97	0.001	0.89
BMD VALUES							
Whole Scan							
Whole body	**0.03**	**0.009**	**0.05**	**-0.013**	**0.01**	*0*.*005*	*0*.*09*
Pelvic	*0*.*06*	-0.008	0.28	-0.001	0.92	**0.008**	**0.03**
Arms (mean)	0.26	-0.004	0.20	0.002	0.52	0.002	0.30
Legs (mean)	**0.00**	**0.028**	**0.00**	**-0.034**	**0.00**	**0.006**	**0.05**
Spine							
Lumbar (mean L_1_–L_4_)	0.83	0.000	0.96	0.003	0.74	-0.003	0.55
Femoral Regions							
Proximal femur (mean)	*0*.*08*	-0.005	0.42	-0.003	0.70	**0.007**	**0.03**
Femoral neck	0.65	-0.005	0.37	0.004	0.51	0.001	0.78
Trochanter	**0.05**	-0.003	0.54	-0.003	0.56	**0.006**	**0.02**
Ward’s triangle	0.99	-0.001	0.88	0.001	0.88	0.000	0.98

All models are adjusted for age, gender, education, marital status, income, BMI, fat mass, lean mass, alcohol intake, smoking, nutritional status, frailty, thyroid disease, arthritis and calcium, by backward elimination (with predictor retained if *p*<0.2). Statistically significant associations (*p*<0.05) are highlighted in bold and the trends are in italics. SB, sedentary behavior; LPA, light physical activity; MVPA, moderate-to-vigorous physical activity.

**Table 5:** Compositional behavior model for bone mass variables for the proportion of the waking hours of day spent in SB, LPA and MVPA for men.

VARIABLE	MODEL *P*-VALUE	γ SB	*P*-VALUE	γ LPA	*P*-VALUE	γ MVPA	*P*-VALUE
BMC VALUES							
Whole Scan							
Whole body	0.13	-40.460	0.10	23.540	0.41	16.920	0.26
Pelvic	0.38	-6.343	0.22	4.575	0.45	1.769	0.54
Arms (mean)	0.10	**-3.784**	**0.05**	2.882	0.20	0.902	0.40
Legs (mean)	**0.04**	-6.185	0.18	0.721	0.89	**5.464**	**0.04**
Spine							
Lumbar (mean L_1_–L_4_)	0.38	-0.437	0.16	0.429	0.24	0.009	0.96
Femoral Regions							
Proximal femur (mean)	0.44	-0.463	0.42	0.100	0.90	0.366	0.30
Femoral neck	0.99	0.009	0.93	-0.015	0.89	0.006	0.91
Trochanter	0.50	-0.130	0.29	0.093	0.51	0.036	0.60
Ward’s triangle	0.21	-0.021	0.16	0.012	0.46	0.008	0.31
BMD VALUES							
Whole Scan							
Whole body	*0*.*08*	-0.009	0.31	-0.002	0.87	**0.011**	**0.04**
Pelvic	**0.01**	**-0.027**	**0.05**	0.010	0.56	**0.017**	**0.02**
Arms (mean)	0.36	-0.007	0.20	0.005	0.42	0.002	0.53
Legs (mean)	**0.03**	-0.016	0.09	0.005	0.67	**0.011**	**0.04**
Spine							
Lumbar (mean L_1_–L_4_)	0.14	*-0*.*028*	*0*.*06*	0.023	0.17	0.005	0.54
Femoral Regions							
Proximal femur (mean)	0.33	-0.009	0.43	0.001	0.93	0.008	0.21
Femoral neck	0.12	*-0*.*019*	*0*.*08*	0.012	0.34	0.007	0.26
Trochanter	0.66	-0.003	0.76	-2.000	0.87	0.004	0.39
Ward’s triangle	0.31	-0.016	0.20	0.010	0.47	0.006	0.42

All models are adjusted for age, education, marital status, income, BMI, fat mass, lean mass, alcohol intake, smoking, nutritional status, frailty, thyroid disease, arthritis and calcium, by backward elimination (with predictor retained if *p*<0.2). Statistically significant associations (*p*<0.05) are highlighted in bold and the trends are in italics. SB, sedentary behavior; LPA, light physical activity; MVPA, moderate-to-vigorous physical activity.

**Table 6:** Compositional behavior model for bone mass variables for the proportion of the waking hours of day spent in SB, LPA and MVPA for women.

VARIABLE	MODEL *P*-VALUE	γ SB	*P*-VALUE	γ LPA	*P*-VALUE	γ MVPA	*P*-VALUE
BMC VALUES							
Whole Scan							
Whole body	**0.03**	**43.228**	**0.01**	**-39.267**	**0.03**	-2.960	0.71
Pelvic	0.38	2.196	0.39	-0.817	0.77	-1.380	0.27
Arms (mean)	**0.03**	-0.971	0.87	**2.517**	**0.05**	**-1.546**	**0.01**
Legs (mean)	**0.00**	**18.586**	**0.00**	**-19.300**	**0.00**	0.712	0.64
Spine							
Lumbar (mean L_1_–L_4_)	0.13	0.190	0.27	-0.052	0.79	-0.139	0.10
Femoral Regions							
Proximal femur (mean)	0.76	0.108	0.79	0.025	0.95	-0.133	0.50
Femoral neck	0.76	-0.043	0.48	0.052	0.47	-0.008	0.81
Trochanter	0.76	-0.048	0.54	0.033	0.70	0.015	0.70
Ward’s triangle	0.36	0.009	0.39	-0.003	0.77	-0.006	0.26
BMD VALUES							
Whole Scan							
Whole body	**0.00**	**0.022**	**0.00**	**-0.023**	**0.00**	0.001	0.79
Pelvic	0.33	0.008	0.35	-0.003	0.75	-0.005	0.25
Arms (mean)	0.79	-0.003	0.52	0.002	0.64	0.001	0.82
Legs (mean)	**0.00**	**0.063**	**0.00**	**-0.069**	**0.00**	0.005	0.18
Spine							
Lumbar (mean L_1_–L_4_)	0.33	0.013	0.21	-0.009	0.45	-0.004	0.42
Femoral Regions							
Proximal femur (mean)	0.90	0.000	0.98	0.002	0.81	-0.002	0.65
Femoral neck	0.62	0.002	0.83	0.002	0.82	-0.004	0.34
Trochanter	0.78	-0.002	0.77	0.000	1.00	0.002	0.53
Ward’s triangle	0.25	0.007	0.40	-0.001	0.90	-0.006	0.15

All models are adjusted for age, education, marital status, income, BMI, fat mass, lean mass, alcohol intake, smoking, nutritional status, frailty, thyroid disease, arthritis and calcium, by backward elimination (with predictor retained if *p*<0.2). Statistically significant associations (*p*<0.05) are highlighted in bold and the trends are in italics. SB, sedentary behavior; LPA, light physical activity; MVPA, moderate-to-vigorous physical activity.

## Discussion

To the best of our knowledge, this investigation is the first to use compositional analysis to examine the associations between the relative distribution of time spent in SB, LPA and MVPA and bone mass variables in the elderly. This novel analytical method allows for a better understanding of how time distribution during the waking day influences the bone mass in this population. Our main novel finding was that the combined effects of these behaviors were significantly associated with leg BMC and BMD, femoral neck BMC and whole body BMD. However, when the sample was stratified we found that these associations were gender specific. Daily movement behavior was associated with leg and pelvic bone mass in the men’s subgroup and, whole body, leg and arm bone mass in women.

Although this is the first study to use compositional analyses to examine these relationships, the independent association between objectively-measured SB and PA with BMD in older adults has been studied previously [27]. A large number of previous studies have examined the association between MVPA and bone health in different populations, but their analyses did not adjust for SB and LPA [16, 27–32]. Likewise, and as our study also indicates, the relationship between BMD and PA should be studied distinguishing by sex, given that this relationship appears to be sex dependent [30, 33].

Despite women just showing a negative association between MVPA and arm BMC, men showed a positive effect of MVPA in leg BMC and BMD, whole body and pelvic BMD. Thus, PA and specially MVPA seem to be a good way to prevent osteoporosis and to improve bone health status in older men; which is now becoming a big issue as before it was largely underdiagnosed. Furthermore, our bone health profiles showed that older men with normal bone health spent more time in LPA and MVPA. This strong relation between bone variables and MVPA has also been previously demonstrated in men, but not in women [27], who did not show apparent differences in bone mass with high or low PA [28]. As previously confirmed, the adhesion to PA guidelines has not been related to femoral neck BMD in older women [29]; however, our study confirms that MVPA had a positive effect both on femoral neck and trochanter when we studied the whole sample. Thus, in the femoral regions, MVPA related to the time spent in the other movement behaviors would be the intensity of activity that may be able to improve the bone mass. Probably, we found benefits with MVPA even if the complete model was not significant because according to the variation matrix this behavior was the least co-dependent. Meanwhile, the LPA effect did not seem to be relevant and clear in men and women; as was recently determined by The Healthy Ageing Initiative [34]. Perhaps the reason for these differences between sex and intensity could be the time spent in these specific behaviors. Various studies demonstrated that women spend more time in LPA and less in MVPA than men [27, 30–32], in the same way as in our sample. Women spent 6.6% more time in LPA (52.9 min/day) and 1.6% less in MVPA (13.5 min/day) than men.

Another possible explanation could be the differences in the modality of exercise performed by men and women [27, 30]. Generally, it has been estimated that in women, PA was divided into three main domains (work, sports, household), with consistently lower levels of strenuous exercise and vigorous work over their lifetimes [30, 35]. In addition, Martyn St-James & Caroll (2006) explained the impact forces associated with walking at light and at moderate-to-vigorous intensity may not have been large enough to adequately preserve BMD, as regular walking is one of the main activities performed by women [36]. According to the Survey of Sports Habits in Spain 2015 [37], the main sport modalities carried out by men were football, cycling, swimming, running and muscle-building, while for women they were cycling, swimming, walking and soft gymnastics. In older people, these specific modalities also predominate; thus, Spanish old men mainly practice osteogenic sport modalities, compared to Spanish old women, who prefer activities that involve low muscle-skeletal tension.

High-impact activities and resistance training are the most widely utilized form of exercise to reduce the burden of osteoporosis and to maintain and increase BMD in older adults [38–44]. Thus, some intervention studies demonstrated that older men and women, who practiced high-impact exercise or high-intensity resistance training, managed to gain or maintain BMD compared to the control group [36, 45–49]. Nonetheless, McMillan et al. (2017) and Beck et al. (2011) highlight that free-living high-impact PA may not be sufficient to generate increases in BMD in older women, considering other important factors in the maintenance of BMD during aging, such as the necessity of improving the geometric response to load [44, 50]. Maybe for these reasons, another study found that older women who did high-impact exercise maintained their BMD equally compared to the control group [51]. It was also demonstrated that whilst high impact may not be positively associated with BMD, moderate and low impacts were inversely associated with spine and hip BMD [39, 52]. Consequently, dividing the sample by the amount of impact against the intensity could be an option to evaluate more specifically the relationship between bone mass and movement behaviors; especially in women, who practice less osteogenic activities and MVPA.

However, geometry and morphology are independently associated with fracture risk, indicating that these characteristics also respond to various forms of PA [44]. All this could clearly explain that older people without bone fractures spent less time in SB and more time in LPA and overall, in MVPA than the entire sample, even in women, where the association related to MVPA and LPA did not show benefits for bone mass. Women without bone fractures presented less time in SB and more time in LPA and MVPA than the entire subgroup. Thus, older women might decrease bone fracture risk through PA despite the fact that PA did not improve either BMC or BMD. Regarding the different profiles for bone health, our results coincided with those found by another author, not detecting differences in PA between women with normal BMD and osteopenic women [32].

Finally, in relation to SB relative to the other movement behaviors, it was negatively associated with arm BMC and pelvic BMD in older men. Unlike the men’s results, in our study SB was positively associated with whole body and leg bone mass in older women. To explain the controversial results that were found in the women’s subgroup, it is essential to know that all our findings are about relative time in SB over the other behaviors and not just time in SB. In other words, if LPA decreases then the ratio will increase inflating the relative time in SB. Moreover, in women there is usually more LPA and less MVPA, which actually occurred in our study as shown in Table 2. Thus, it is possible that the relationship is driven by the ratio between rest and LPA rather than purely the SB time. According to this, our results would go against the well known advice that just being on your feet is good for BMD. Another related explanation could be that more SB probably means also more bouts of SB which increases the frequency of breaks in SB (sit-to-stand), which is positively associated with BMD [27, 29]. Likewise, a last explanation could be that women with higher BMD also do more MVPA and therefore need more rest (SB). Therefore, more MVPA + more SB and less LPA would also increase the ratio SB/(LPAxMVPA) because the product LPAxMVPA will be lower suggesting slightly more SB.

Our study is not without limitations. We did not record objective measures of sleep, or sleep quality. Nevertheless, this information has not been made available in other studies [15, 17, 53] either, and we did objectively measure the waking hours. Similarly, although accelerometry is an objective and validated method, it may not detect differences between sitting and standing positions, and may overestimate sedentary time [54]. Furthermore, as with all cross-sectional analysis, causal inference is limited and the estimated effects reflect population shift in distribution of time [15]. Nonetheless, a main strength of our study stems from the fact that it is the first to include the novel analytical approach to deal with all waking hour data, assessing a relatively large cohort of older people with objectively assessed PA, body composition, bone health and health indicators. In addition, we used the gold standard DXA method to assess the body composition and bone mass and, what is more, we differentiated between subgroups (sex, bone fractures and bone health), which has been requested previously [53]. In addition, we excluded all the DXA scans with metal prosthesis or similar artifacts, which could affect the actual results of the bone mass [22, 23]. Finally, all variables that could have an impact on bone mass were included in the analysis as covariates to ensure that our results were not influenced by any factor (e.g., diet, smoking, frailty, etc.) [55].

## Conclusion

In general, the positive effect of MVPA on bone mass is the clearest effect of PA in older people; however, this relation is greater in men than women. Therefore, we identified that to increase MVPA, and to maintain or to decrease time spent in LPA and SB, contributes toward a more favorable bone mass, overall in older men. In addition, our findings also support the importance of PA in women despite the fact that MVPA and LPA do not show a visible benefit in bone mass. Thus, older women might decrease bone fracture through PA increase and the reduction of time spent in SB. Longitudinal research is required to confirm the causality of the relationships observed in the current study, as well as compositional analysis differentiating between the amount of impact or dividing by stages of frailty that could represent a significant advance in knowledge.
